# Alterations in bone turnover markers in patients with noncardio-embolic ischemic stroke

**DOI:** 10.1371/journal.pone.0207348

**Published:** 2018-11-29

**Authors:** K. Mathold, P. Wanby, L. Brudin, S. P. Von, M. Carlsson

**Affiliations:** 1 Department of Geriatric Medicine, County Hospital of Kalmar, Kalmar, Sweden; 2 Section of Endocrinology, Department of Internal Medicine, County Hospital of Kalmar, Kalmar, Sweden; 3 Department of Clinical Physiology, County Hospital of Kalmar, Kalmar, Sweden; 4 Department of Clinical Microbiology and Infectious Diseases, County Hospital of Kalmar, Kalmar, Sweden; 5 Department of Clinical Chemistry, County Hospital of Kalmar, Kalmar, Sweden; 6 Department of Medicine and Optometry, Linnaeus University, Kalmar, Sweden; Garvan Institute of Medical Research, AUSTRALIA

## Abstract

**Background:**

The major cause of ischemic stroke is unstable or thrombogenic atherosclerotic plaques. Vascular calcification, a process that appears crucial for plaque stability, shares common features with bone formation. Many bone turnover proteins exhibit metabolic properties, but the evidence is conflicting regarding their possible involvement in vascular disease. Antibodies against sclerostin and dickkopf-1 are currently being evaluated as potential therapy for treating bone disorders. It is important to carefully assess the cardiovascular and metabolic effects of these proteins. The aim of the present study was to explore serum levels of bone turnover markers in patients with acute noncardio-embolic ischemic stroke in comparison with healthy controls.

**Methods:**

In a cross-sectional study, we compared 48 patients aged ≥75 years with noncardio-embolic ischemic stroke and 46 healthy controls. Serum levels of dickkopf-1, sclerostin, osteoprotegerin, osteopontin and osteocalcin were determined by Luminex technique.

**Results:**

We found clearly increased serum levels of osteoprotegerin, sclerostin, dickkopf-1 and osteopontin in patients with stroke compared with healthy controls. No difference was seen in serum levels of osteocalcin between the two groups.

**Conclusion:**

Our findings strengthen the hypothesis of bone turnover markers being involved in vascular disease. Whether these proteins can be used as candidate markers for increased stroke risk or prognostic biomarkers remains to be further elucidated.

## Introduction

Arterial occlusion secondary to atherosclerosis is believed to account for 80% of all ischemic strokes [1]. The pattern of calcification of atherosclerotic plaques, i.e. micro- or macrocalcifications, appear to be crucial for plaque stability [2–3]. It is well known that vascular calcification is an actively regulated process, which exhibits common features with bone formation [4–5]. Cells resembling osteoblasts and osteoclasts are found in atherosclerotic lesions, and many cell types including pericytes, vascular smooth muscle cells, and macrophages are capable of this osteochondrogenic differentiation [3,5]. Several biomarkers have been examined for predictors of increased risk for plaque instability. For example, non-collagenous bone matrix proteins, such as osteocalcin (OCN) and osteopontin (OPN), have been found to co-localize with calcium deposits in atherosclerotic lesions according to previous studies [6–7]. However, further research is needed to explore the hypothesis of common mechanisms in bone metabolism and vascular disease. We have chosen to focus on osteoprotegerin (OPG), OCN, dickkopf-1(DKK1), sclerostin and OPN, which are markers of bone turnover [8] but exhibit metabolic properties as well. The aim of the present study was to explore serum levels of these biomarkers in patients with acute noncardio-embolic ischemic stroke in comparison with healthy controls.

## Materials and methods

### Study population

The current study is part of a larger study regarding serum levels of vitamin D in patient populations at risk for vitamin D deficiency [9]. All participants were from the same geographical area (Kalmar County in southeastern Sweden). Patients over the age of 74 were included, and an age-matched control group of persons considering themselves to be in good health, was created. Recruitment of patients and healthy controls took place between February 2014 and April 2015. Forty-eight patients with acute noncardio-embolic stroke were included consecutively after admission to the stroke unit at the county hospital of Kalmar, Sweden. Eight patients with cerebral haemorrhage and 14 patients with ischaemic stroke in the presence of atrial fibrillation were also included, but studied separately. Patients were diagnosed with acute stroke according to guidelines for ordinary clinical routine. Diagnostic evaluation, including brain computed tomography (CT) and 12-lead electrocardiogram, was performed on all patients. Only one patient was diagnosed with significant, symptomatic carotid stenosis and subjected to carotid surgery. Fifteen patients with stroke had ongoing medication with statins, 22 patients were already on platelet aggregation inhibitors, and five patients took diabetes medication at baseline. Only four patients were current smokers. Thirty-six stroke patients took hypertension- or heart medication at baseline. Eight patients with stroke had osteoporosis according to their journals. Five were taking calcium and vitamin D supplements. None of the patients had ongoing treatment with bisphosphonates, but one patient was taking denosumab.

After advertising in the local newspaper, at supermarkets, or at pensioner´s associations, volunteering healthy controls (age≥75 years) were invited to participate in the study. All volunteers considered themselves in excellent health. A nurse interviewed all patients and healthy controls regarding medical history and ongoing medication upon enrolment. This patient data was also collected via electronic medical records. All participants underwent anthropometric measurement and biochemical screening on the same occasion. Blood pressure was measured as well as height and weight. Body mass index (BMI) was calculated. Waist circumference was measured midway between the lowest ribs and the iliac crest. Dementia, diabetes medication, previous or present stroke, or fragility fracture, were considered exclusion criteria. Additional exclusion criteria were applied to limit inclusion of controls with atherosclerotic disease. People with a history of vascular events, ongoing medication with any anticoagulant, antihypertensive medication, lipid lowering agent, medication for any heart condition, or antidiabetic medication, were therefore excluded. Current smokers were also excluded, as well as those with B-HbA1c ≥48 mmol/mol. Forty-six healthy controls remained after implementation of these additional exclusion criteria. More men than women exhibited manifest atherosclerotic disease, therefore only 14 men were included in the current control group. Baseline characteristics in the 48 patients with noncardio-embolic stroke, and in the 46 healthy controls, are shown in Table 1.

**Table 1:** Baseline characteristics in patients with stroke (n = 48) and healthy controls (n = 46).

Parameter	Stroke	Healthy controls	Difference (p-value)	
			Crude	Adjusted*
**N**	48	46		
**Age (years)**				
**Mean (SD)**	83 (5.7)	77 (2.2)		
**Median (range)**	83 (75–97)	77 (74–84)	<0.001	-
**Gender (n; %)**				
**Male**	24 (50)	14 (30)		
**Female**	24 (50)	32 (70)	0.084	-
**Smokers (n; %)**				
**Not current**	37 (77.1)	46 (100)		
**Current**	4 (8.3)	0 (0)	0.043	-
**Missing data**	7 (14.6)	0 (0)		
**BMI (kg/m**^**2**^**)**				
**Mean (SD)**	24.7 (4.0)	24.3 (3.5)		
**Median (range)**	25 (16–32)	24 (16–35)	0.660	-
**Waist (cm)**				
**Mean (SD)**	95.4 (13.7)	84.9 (10.1)		
**Median (range)**	96 (69–123)	87 (64–104)	<0.001	-
**Systolic blood pressure (mmHg)**				
**Mean (SD)**	155 (21)	129 (14)		
**Median (range)**	157 (95–195)	130 (93–160)	<0.001	-
**Diastolic blood pressure (mmHg)**				
**Mean (SD)**	77 (14)	69 (8)		
**Median (range)**	80 (40–110)	70 (60–85)	0.001	-
**P-Calcium (mmol/L)**				
**Mean (SD)**	2.33 (0.11)	2.29 (0.06)		
**Median (range)**	2.3 (2.1–2.7)	2.3 (2.1–2.4)	0.042	0.25
**S-Ca ionized (mmol/L)**				
**Mean (SD)**	1.27 (0.06)	1.25 (0.03)		
**Median (range)**	1.3 (1.2–1.5)	1.3 (1.2–1.4)	0.121	0.45
**P-Phosphate (mmol/L)**				
**Mean (SD)**	1.20 (0.19)	1.26 (0.13)		
**Median (range)**	1.2 (0.8–1.7)	1.3 (1.0–1.5)	0.085	0.66
**P-Creatinine (μmol/L)**				
**Mean (SD)**	94.1 (39.3)	75.8 (13.3)		
**Median (range)**	86 (53–255)	77 (49–114)	0.004	-
**Glomerolus filtration rate (eGFR, mL/min/1.73m2)**				
**≤30**	2 (4)	0 (0)		
**30–60**	15 (31)	7 (15)		
**>60**	31 (65)	39 (85)	0.005	-
**S-25-hydroxyvitamin (nmol/L)**				
**Mean (SD)**	62 (28)	76 (27)	0.013	0.20
**Median (range)**	59.0 (16.0–125.0)	74.5 (22.0–154.0)		
**S-PTH (pmol/L)**				
**Mean (SD)**	6.9 (3.9)	6.0 (1.6)		
**Median (range)**	5.8 (2.8–20.0)	5.9 (3.4–9.0)	0.371**	0.79
**B-HbA1c mmol/mol)**				
**Mean (SD)**	43.7 (12.2)	37.0 (2.6)		
**Median (range)**	40 (31–103)	37 (30–44)	<0.001	0.035
**Diabetes medication**				
**No**	43 (90)	46 (100)		
**Yes**	5 (10)	0 (0)	0.056	-
**Hypertension or heart medication**				
**No**	12 (25)	46 (100)		
**Yes**	36 (75)	0 (0)	<0.001	-
**Statin medication**				
**No**	33 (69)	46 (100)		
**Yes**	15 (31)	0 (0)	<0.001	-

* Adjusted for age, sex, waist and kidney function (GFR_category)

** The natural logarithm of PTH was used

Informed consent was obtained from all study participants and documented in their electronic medical records (Cambio COSMIC, Cambio Healthcare Systems, Sweden). For stroke patients with cognitive impairment or aphasia, informed consent was acquired in consultation with their families. The Regional Ethical Review Board Committee of Linköping, Sweden approved the study (Dnr 2013/404-31).

### Assays

Blood samples were collected upon diagnosis with acute stroke and enrolled in the study (within 72 hours). Blood was drawn early in the morning on the hospital ward. Blood samples from healthy controls were collected, either in the morning or afternoon. Both patients and healthy controls were non-fasting. All blood samples were analysed at the Kalmar County Clinical Chemistry Hospital Laboratory according to the manufacturer´s protocol. The bone turnover markers were analysed using xMAP technology with multiplex beads according to Merck Millipore´s instructions. Luminex´s xMAP instrument MagPix LX 200 (Luminex, Austin, TX, USA) and plates from Merck Millipore (Human Bone Magnetic Bead Panel) were used. All samples were analysed in duplicate, and if differing by more than 20%, measurements were repeated on a second aliquot. Coefficients of variation (% CVs) for low and high concentrations, respectively, in the control material were <5% for all bone turnover markers. An electrochemiluminescence immunoassay was used to measure serum PTH (Cobas e 601, Roche Diagnostics, Basel, Switzerland). Plasma phosphate, calcium and creatinine were measured by a colorimetric method (Vitros 5.1 FS, Ortho Clinical Diagnostics, Rochester, NY, USA). Measurement of B-HbA1c was made with Cobas c501 (Roche Diagnostics, Basel, Switzerland). The estimated glomerulus filtration rate (eGFR) was calculated using the MDRD formula. We used total serum 25-hydroxyvitamin D (25-OH-vitamin D2 + 25-OH-vitamin D3) as an indicator of vitamin D status, which was measured by tandem mass spectrometry (LC-MS/MS) using the API 4000 instrument with an atmospheric pressure chemical ionization (APCI) source (AB Sciex, Concord, ON, Canada) and UFLC Schimadzu Prominence (Schimadzu, Kyoto, Japan). The calibrators used were traceable to the National Institute of Standards and Technology (NIST), standard reference material (SRM) 972a, Vitamin D in Human Serum.

### Statistical analysis

The natural logarithms of all bone turnover markers except OCN were applied. One-way ANOVA was used to compare mean serum levels of bone turnover markers between stroke patients and healthy controls. Duncan’s post hoc test was applied for further analysis. Differences in the serum levels of bone turnover markers between the two groups were adjusted for age, sex, waist circumference, and kidney function (eGFR) by analysis of co-variance (ANCOVA). Correlation coefficients were determined for bone turnover markers in relation to orienting blood samples and anthropometric measurements. STATISTICA (version 12, Statsoft, Tulsa, USA) was used for all statistical analyses. P-values < 0.05 were considered significant.

## Results

Serum levels of sclerostin, DKK1, OPG and OPN were significantly higher in patients with stroke compared with controls. We found no significant difference in serum levels of OCN between the two groups (Table 2). Adjustment was made for age, sex, waist circumference, and kidney function (eGFR) regarding all bone turnover markers.

**Table 2:** Bone turnover markers in patients with stroke (n = 48) and healthy controls (n = 46).

Parameter	Stroke	Healthy controls	Difference (p-value)	
			Crude	Adjusted*
**N**	48	46		
**S-Sclerostin (μg/L)**				
**Geom mean**	3.0	1.9		
**LnSD**	0.49	0.57		
**Median (range)**	3.0 (1.0–7.3)	2.0 (0.2–6.5)	<0.001	0.00
**S-Dickkopf-1 (ng/L)**				
**Geom mean**	251	163		
**LnSD**	0.40	0.45		
**Median (range)**	275 (96–513)	172 (63–467)	<0.001	0.00
**S-Osteoprotegerin (ng/L)**				
**Geom mean**	435	188	<0.001	<0.001
**LnSD**	0.42	0.40		
**Median (range)**	420 (224–2 302)	201 (69–351)		
**S-Osteopontin (μg/L)**				
**Geom mean**	32.6	19.5	<0.001	0.01
**LnSD**	0.57	0.41		
**Median (range)**	38 (8–98)	21 (4–40)		
**S-Osteocalcin (μg/L)**				
**Mean (SD)**	8.6 (4.8)	10.1 (3.2)	0.077	0.12
**Median (range)**	8 (2–27)	10 (5–18)		

*) Adjusted for age, sex, waist, and kidney function (GFR_category)

**Fig 1:**
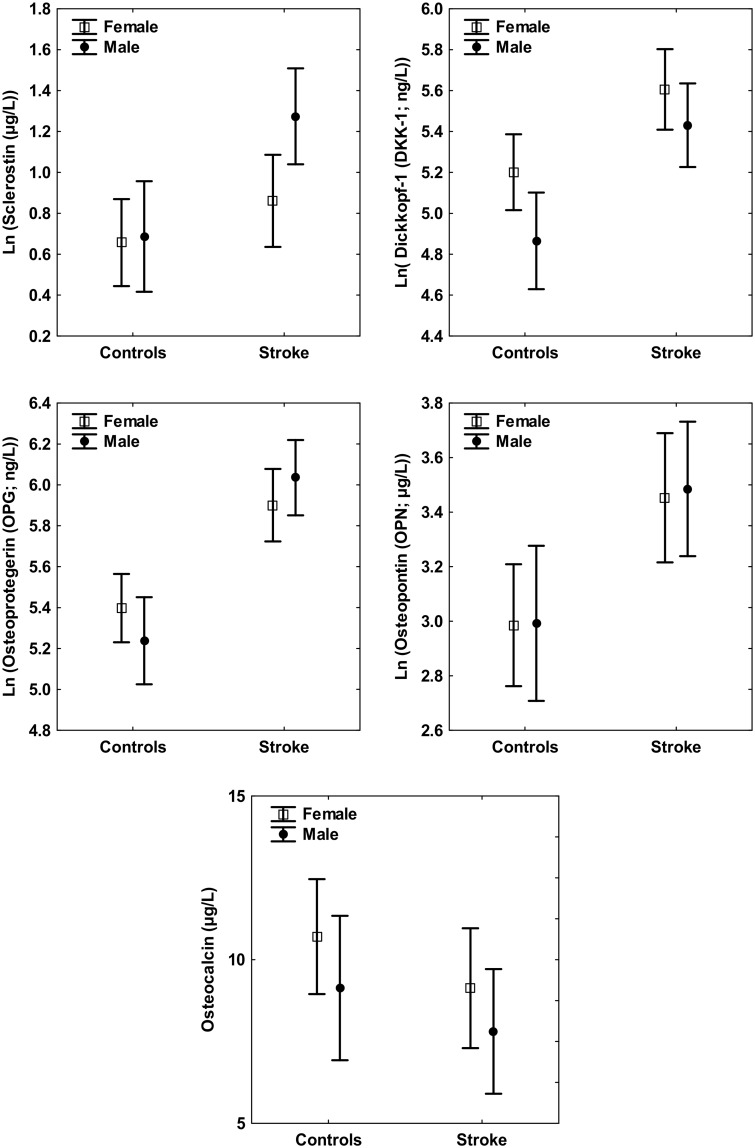
Bone turnover markers in male and female patients with stroke (n = 48) and healthy controls (n = 46).

A gender difference was observed only for serum levels of sclerostin. Men with stroke exhibited higher levels of sclerostin compared to women. No gender difference was observed in the control group for any of these bone turnover markers (Fig 1). A correlation was seen between renal function parameters (plasma creatinine and eGFR) and two of the biomarkers (sclerostin and OCN) in patients with stroke, but no such correlation was observed in the control group. No correlation was demonstrated between blood HbA1c and these bone turnover markers, neither for stroke patients nor controls. Anthropometric measurements such as BMI, waist circumference, and blood pressure were measured. Only OPG in stroke patients was found to correlate with BMI. Sclerostin in stroke patients was found to correlate with waist circumference. Regarding blood pressure, a correlation was demonstrated only between systolic blood pressure and sclerostin in controls. A few correlations were demonstrated between these bone turnover markers and bone-related laboratory parameters such as serum PTH, plasma calcium, and plasma phosphate. For example, PTH levels were found to correlate with DKK1, OPN, and OCN in controls. A correlation was also seen between ionized calcium concentrations and sclerostin or DKK1 in controls. Several correlations were demonstrated between bone turnover markers in the control group. For example, a positive correlation was seen between sclerostin and DKK1, sclerostin and OPG, and between DKK1 and OPG in controls. In the current study serum 25(OH)D concentration was not found to be strongly associated with any of the bone turnover markers, neither in healthy controls nor in stroke patients (Table 3).

**Table 3:** Cross correlation tables calculated for the two groups separately.

Stroke	LnSclerostin	LnDickkopf-1	LnOsteoprotegerin	LnOsteopontin	Osteocalcin
**Age**	*-0*.*33 (p = 0*.*023)*	-0.17 (p = 0.244)	**0.40 (p = 0.004)**	0.05 (p = 0.730)	0.00 (p = 1.00)
**Gender**	***0*.*60 (p<0*.*001)***	-0.10 (p = 0.485)	0.06 (p = 0.705)	0.01 (p = 0.950)	-0.09 (p = 0.555)
**BMI**	0.26 (p = 0.079)	0.15 (p = 0.311)	*-0*.*33 (p = 0*.*021)*	0.03 (p = 0.858)	-0.06 (p = 0.689)
**Waist**	*0*.*33 (p = 0*.*022)*	0.10 (p = 0.520)	-0.17 (p = 0.247)	-0.02 (p = 0.886)	-0.13 (p = 0.366)
**SBP**	-0.14 (p = 0.340)	0.13 (p = 0.371)	0.01 (p = 0.972)	0.01 (p = 0.955)	0.16 (p = 0.269)
**DBP**	0.15 (p = 0.302)	-0.12 (p = 0.436)	-0.09 (p = 0.552)	-0.08 (p = 0.570)	0.25 (p = 0.085)
**Calcium**	0.13 (p = 0.396)	0.25 (p = 0.087)	-0.08 (p = 0.582)	-0.21 (p = 0.151)	-0.11 (p = 0.448)
**Ca ionized**	0.19 (p = 0.186)	0.20 (p = 0.164)	0.02 (p = 0.887)	-0.07 (p = 0.638)	-0.03 (p = 0.852)
**Phosphate**	-0.06 (p = 0.685)	-0.03 (p = 0.840)	-0.04 (p = 0.797)	0.08 (p = 0.610)	**0.38 (p = 0.008)**
**Creatinine**	***0*.*54 (p<0*.*001)***	0.14 (p = 0.351)	0.27 (p = 0.067)	0.17 (p = 0.259)	**0.37 (p = 0.010)**
**eGFR**	*-0*.*34 (p = 0*.*019)*	-0.13 (p = 0.363)	-0.22 (p = 0.141)	-0.09 (p = 0.538)	*-0*.*30 (p = 0*.*039)*
**25(OH)D**	0.19 (p = 0.187)	-0.12 (p = 0.424)	-0.04 (p = 0.807)	-0.05 (p = 0.743)	0.09 (p = 0.553)
**PTH**	0.07 (p = 0.661)	0.23 (p = 0.112)	0.01 (p = 0.946)	0.24 (p = 0.101)	**0.42 (p = 0.003)**
**HbA1c**	-0.02 (p = 0.874)	0.22 (p = 0.138)	0.17 (p = 0.259)	-0.03 (p = 0.848)	0.06 (p = 0.677)
**LnSclerostin**		0.08 (p = 0.582)	0.22 (p = 0.136)	0.02 (p = 0.887)	0.17 (p = 0.240)
**LnDickkopf-1**			-0.12 (p = 0.426)	-0.10 (p = 0.482)	0.01 (p = 0.953)
**LnOsteoprotegerin**				0.19 (p = 0.205)	0.16 (p = 0.270)
**LnOsteopontin**					*0*.*33 (p = 0*.*020)*
**Osteocalcin**					-
**Controls**					
**Age**	-0.08 (p = 0.618)	0.18 (p = 0.224)	0.08 (p = 0.584)	0.02 (p = 0.885)	-0.03 (p = 0.852)
**Gender**	0.12 (p = 0.427)	-0.28 (p = 0.061)	-0.23 (p = 0.132)	-0.02 (p = 0.885)	-0.21 (p = 0.158)
**BMI**	0.17 (p = 0.268)	-0.10 (p = 0.494)	-0.05 (p = 0.764)	-0.28 (p = 0.062)	-0.07 (p = 0.669)
**Waist**	0.22 (p = 0.148)	-0.14 (p = 0.363)	-0.15 (p = 0.336)	-0.19 (p = 0.195)	-0.16 (p = 0.280)
**SBP**	**0.38 (p = 0.010)**	0.11 (p = 0.486)	0.22 (p = 0.139)	0.05 (p = 0.747)	-0.02 (p = 0.915)
**DBP**	0.11 (p = 0.465)	-0.13 (p = 0.400)	0.02 (p = 0.876)	0.15 (p = 0.336)	0.09 (p = 0.547)
**Calcium**	0.16 (p = 0.282)	*0*.*36 (p = 0*.*014)*	0.12 (p = 0.430)	-0.06 (p = 0.680)	-0.22 (p = 0.151)
**Ca ionized**	*0*.*30 (p = 0*.*046)*	*0*.*33 (p = 0*.*024)*	0.22 (p = 0.149)	-0.05 (p = 0.723)	-0.17 (p = 0.268)
**Phosphate**	-0.02 (p = 0.886)	0.11 (p = 0.461)	0.04 (p = 0.785)	0.13 (p = 0.373)	0.26 (p = 0.080)
**Creatinine**	0.06 (p = 0.711)	-0.13 (p = 0.394)	0.01 (p = 0.922)	0.00 (p = 0.991)	-0.06 (p = 0.711)
**eGFR**	0.11 (p = 0.476)	-0.03 (p = 0.860)	-0.02 (p = 0.902)	0.17 (p = 0.266)	-0.23 (p = 0.123)
**25(OH)D**	0.12 (p = 0.408)	0.24 (p = 0.108)	0.24 (p = 0.103)	-0.08 (p = 0.605)	-0.11 (p = 0.462)
**PTH**	-0.18 (p = 0.219)	*-0*.*32 (p = 0*.*029)*	-0.18 (p = 0.237)	*0*.*30 (p = 0*.*040)*	**0.44 (p = 0.003)**
**HbA1c**	-0.01 (p = 0.966)	-0.13 (p = 0.407)	-0.22 (p = 0.138)	-0.28 (p = 0.058)	-0.13 (p = 0.379)
**LnSclerostin**		**0.40 (p = 0.006)**	***0*.*53 (p<0*.*001)***	-0.09 (p = 0.540)	-0.17 (p = 0.265)
**LnDickkopf-1**			***0*.*57 (p<0*.*001)***	0.00 (p = 0.993)	-0.09 (p = 0.561)
**LnOsteoprotegerin**				0.11 (p = 0.477)	0.04 (p = 0.798)
**LnOsteopontin**					***0*.*58 (p<0*.*001)***
**Osteocalcin**					-

To learn more about serum levels of these biomarkers in patients with stroke of different subtypes, blood samples were also collected in a small number of patients with cerebral hemorrhage (n = 8), and in patients with ischemic stroke in the presence of atrial fibrillation (n = 14). No difference was found in serum levels of sclerostin, DKK1, OPG, and OPN between stroke subtypes. A small difference was found in levels of osteocalcin in patients with noncardio-embolic stroke compared with patients with ischemic stroke in the presence of atrial fibrillation (p = 0.04). Lowest levels of osteocalcin were found in patients with ischemic stroke and atrial fibrillation.

## Discussion

In this cross-sectional study of patients with acute noncardio-embolic ischemic stroke, we investigated serum levels of five proteins usually regarded as bone turnover markers. There is growing evidence for a connection between these biomarkers and various diseases, such as diabetes and atherosclerosis, in addition to bone disease. Interestingly enough, in the current study we saw large differences in stroke patients compared to controls regarding all bone turnover markers except for OCN. Serum levels of OPG, OPN, DKK1, and sclerostin were much higher in stroke patients compared to controls. However, no significant differences were observed in serum levels of OCN between the two groups. To the best of our knowledge this is the first study simultaneously investigating all of these biomarkers in stroke patients.

### Sclerostin and Dickkopf-1

We found increased serum levels of both DKK1 and sclerostin in patients with acute noncardio-embolic ischemic stroke. DKK1 and sclerostin are two negative regulators of the canonical Wnt signalling pathway. This signalling pathway is involved in a wide range of fundamental processes such as embryonic development, cell differentiation, angiogenesis, inflammation, and apoptosis [10–12]. More recently, alterations in Wnt signalling have also been demonstrated in atherosclerosis [13–14] and diabetes mellitus [15–16].

Sclerostin, which is a glycoprotein encoded by the *SOST* gene, inhibits osteoblast activity, and is mainly expressed in osteocytes [17–19]. Phase II studies of the monoclonal antibody romosozumab, which binds to sclerostin, have shown increased bone mineral density in postmenopausal women [17]. However, *in vitro* assays have also confirmed expression of sclerostin in vascular smooth muscle cells, which had been transformed into mineralizing osteoblast-like cells. There are a few studies suggesting that hyperglycaemia facilitates or induces this differentiation, since circulating levels of sclerostin seem to be increased in atherosclerotic disease in Type 2 diabetes mellitus [20]. Interestingly we found no correlation between HbA1c and any of the bone turnover markers including sclerostin in the present study. We did find a correlation between plasma creatinine, eGFR and sclerostin in stroke patients. Vascular calcification is a well-known complication in patients with end-stage renal failure [21]. Interestingly creatinine did not correlate with sclerostin in healthy controls.

DKK1 is involved in embryonic development [22] and also disrupts osteoblast activity and differentiation [23]. The administration of anti-DKK1 monoclonal antibodies has resulted in improved bone mineral density when tested on mice [24]. DKK1 has also been considered to play an important role in the development of atherosclerosis [25–26]. Interestingly, plasma levels of DKK1 seem to increase with increasingly unstable coronary disease [25]. In a study performed by Seifert-Held and co-workers, plasma levels of DKK1 were also significantly higher in patients with acute ischemic stroke, compared to patients with stable cerebrovascular disease, and healthy controls [27]. DKK1 has therefore been proposed to play a part in platelet-dependent endothelial activation and platelet-mediated inflammation upon plaque destabilization [21, 28]. In the current study the levels of circulating DKK1 were significantly higher in patients with stroke compared with controls.

To the best of our knowledge only one study regarding sclerostin in patients with stroke has been published previously [29]. In our study, we have been able to reproduce the findings of the study performed by He and colleagues [29], i.e. serum levels of both sclerostin and DKK1 are increased in patients with acute ischaemic stroke. In patients with stroke a gender difference in serum levels of sclerostin was observed, meriting further investigation. Our findings regarding DKK1 and sclerostin are of interest not least when considering the ongoing development of antibodies against both these Wnt signalling antagonists.

### Osteoprotegerin

In our study, we found higher serum levels of OPG in stroke patients compared with controls. OPG is a member of the tumour necrosis factor (TNF) receptor superfamily [30]. It is a soluble protein, which is found in bone tissue but can also be secreted by endothelial cells, vascular smooth muscle cells and pericytes [3]. Several studies demonstrate an association between OPG and large vessel atherosclerosis [31–33]. OPG has also been proposed to affect plaque stability, possibly via modulation of matrix metalloproteinase 9 [34].

There are a few studies of OPG in patients with stroke [30, 33]. For example, Song et al examined circulating OPG levels in patients with stroke of different subtypes. OPG was highest in patients with large artery atherosclerosis and in patients with stroke of two or more causes. Patients with highest OPG levels had the poorest outcome at follow-up after 3 months according to this study [30].

We have demonstrated higher serum levels of OPG in patients with stroke, which is in line with the results from Song among others. There is interesting data from previous studies regarding possible involvement of OPG in plaque stability. Repeated measurements of OPG in patients after stroke might be of value to further address this aspect of vascular disease.

### Osteopontin

Serum levels of OPN were higher in stroke patients compared to controls in our study. OPN is an extracellular matrix protein, believed to participate in many biological processes such as bone turnover, tumour genesis, inflammation, immune response, and wound healing [35–36]. OPN is also thought to be involved in vascular remodelling [37–38]. Higher levels of OPN have been demonstrated in injured arteries in ischaemic lesions or in atherosclerotic plaques compared with normal vessels [39–40]. There are also studies supporting a relationship between OPN and plaque destabilization [37, 41].

Our results of elevated serum levels of OPN in patients with acute ischaemic stroke confirm the findings presented by Carbone and co-workers in 2015. Moreover, Carbone et al reported that serum levels of OPN reach a peak at day seven after an ischemic stroke, and are positively correlated with ischemic lesion volume and poorer neurological scores [39]. Since the design of our study was cross-sectional, no follow-up was made allowing for OPN to be examined in relation to prognosis.

### Osteocalcin

In the current study, we found no significant difference in serum levels of osteocalcin between patients with acute noncardio-embolic stroke and controls. OCN is a non-collagen bone matrix protein, which is expressed by osteoblasts. Serum concentration of OCN is a marker of bone formation [42]. OCN is also expressed in platelets and released upon platelet activation according to a study performed by Foresta et al [43]. The OCN content in platelets is higher in patients with carotid artery occlusive disease, high numbers of cardiovascular risk factors, and in older patients according to the same study. The results from studies regarding OCN and atherosclerosis are, nevertheless, divergent. According to Pennisi and colleagues patients with serious atherosclerotic involvement of the femoral artery exhibit lower serum levels of OCN [44].

In the current study, we found no significant difference in serum levels of osteocalcin in patients with acute noncardio-embolic ischemic stroke compared with controls. Serum levels of OCN were found to be lowest in patients with ischemic stroke in the presence of atrial fibrillation. However, only 14 patients were included in this group. Larger studies in patients with stroke of different subtypes are needed to confirm or reject our findings. OCN has been associated with diabetes in several studies, but interestingly, we found no association between OCN and HbA1c or anthropometric markers in our study. Statins might increase circulating levels of OCN [45], which possibly affects our results. One third of patients with stroke, but none of the healthy controls, had ongoing medication with statins at baseline. Cigarette smoking possibly lowers the levels of OCN [46] but only four patients in our study were current smokers.

The present study is afflicted by some limitations, in part due to its cross-sectional design. Several of these biomarkers might be altered with increasing destabilization of atherosclerotic disease according to previous studies. Our study does not allow for such discrimination, as only patients with acute stroke were examined, and not patients with stable cerebrovascular disease. Another weakness of our study is the limited information of bone health in our stroke patients. Ideally Dual-energy X-ray absorptiometry (DXA) should have been performed to address any influence of osteoporosis on our results. Our findings are also based on a limited number of patients and controls. Larger studies in patients with different subtypes of stroke would be of value to ensure the validity of our findings. It would also be of interest to explore the generalizability to individuals of other ethnic groups and ages, since all our patients and controls were older adults of northern European descent. Unfortunately it was beyond the scope of this study to examine the severity of stroke in relation to bone turnover markers, which is a limitation of the study. Bone turnover markers might be of prognostic value in acute cerebrovascular disease but larger prospective studies are needed.

According to previous studies, several bone turnover markers are also markers of atherosclerotic plaque instability. It is therefore plausible to assume that the levels of many of these bone turnover markers might be affected by statin therapy. There are a few studies indicating a relationship between statins and these biomarkers [47–49], but our study was not designed to address this interesting question. Many of our patients with stroke also had ongoing medication against hypertension or heart conditions. Only five patients had diabetes medication. All of these medicines indicate a high likelihood of atherosclerosis, but we cannot rule out that the levels of bone turnover markers are affected by the medication itself. There are currently no convincing studies addressing this question. Nor is it known whether there is a diurnal variation in circulating bone turnover markers. In our study blood samples were drawn either in the morning or afternoon.

Despite these limitations our study has several important strengths. It is the first to explore these five bone turnover markers simultaneously in patients with acute stroke. Aberrant levels of several bone turnover markers in the same study strengthen the hypothesis of bone turnover mechanisms being involved in vascular disease. Previous studies regarding metabolic aspects of these biomarkers are few and inconclusive. In our study we have been able to confirm or reject several findings from previous studies. We also reached strongly significant statistical results despite a small study sample.

## Conclusion

The main finding of this cross-sectional study in stroke patients is altered serum levels of proteins traditionally regarded as bone turnover markers. Our results also support the theory of these proteins as biomarkers of vascular disease. In the near future antibodies against sclerostin will be on the market as a new therapeutic approach in treating osteoporosis. Further research is needed regarding extraskeletal effects of bone turnover markers, especially when considering possible side effects of these antibodies. The nature of our study is observational, and we are unable to draw any conclusions regarding the causality of our findings. Further studies on a molecular level are needed to learn more about the pathophysiology in atherosclerosis and plaque stability. These serum proteins might also serve as diagnostic tools in acute cerebrovascular disorders, but larger studies are required to confirm our findings and explore appropriate reference intervals. Prospective studies would also be desirable to evaluate the predictive value of these biomarkers in assessing the risk of vascular events in patients with atherosclerosis.
